# Plant growth promoting rhizobacteria and biochar production from *Parthenium hysterophorus* enhance seed germination and productivity in barley under drought stress

**DOI:** 10.3389/fpls.2023.1175097

**Published:** 2023-06-08

**Authors:** Farrukh Gul, Irfan Ullah Khan, Susan Rutherford, Zhi-Cong Dai, Guanlin Li, Dao-Lin Du

**Affiliations:** ^1^ School of Emergency Management, Jiangsu University, Zhenjiang, China; ^2^ School of the Environment and Safety Engineering, Jiangsu University, Zhenjiang, China; ^3^ Department of Botany, Pir Mehr Ali Shah-Arid University (PMAS), Rawalpindi, Pakistan; ^4^ Key Laboratory of Environmental Biotechnology, Research Center for Eco-Environmental Sciences, Chinese Academy of Sciences, Beijing, China; ^5^ Institute of Environmental Health and Ecological Security, School of Environment and Safety Engineering, Jiangsu University, Zhenjiang, China; ^6^ Division of Environmental Science and Ecological Engineering, Korea University, Seoul, Republic of Korea; ^7^ Jiangsu Collaborative Innovation Center of Technology and Material of Water Treatment, Suzhou University of Science and Technology, Suzhou, China

**Keywords:** tolerance, physicochemical, water stress, seed germination, soil nutrients

## Abstract

Drought stress can significantly affect plant growth and development. Biochar (BC) and plant growth-promoting rhizobacteria (PGPR) have been found to increase plant fertility and development under drought conditions. The single effects of BC and PGPR in different plant species have been widely reported under abiotic stress. However, there have been relatively few studies on the positive role of PGPR, BC, and their combination in barley (*Hordeum vulgare* L.). Therefore, the current study investigated the effects of BC from *Parthenium hysterophorus*, drought tolerant PGPR (*Serratia odorifera*), and the combination of BC + PGPR on the growth, physiology, and biochemical traits of barley plants under drought stress for two weeks. A total of 15 pots were used under five treatments. Each pot of 4 kg soil comprised the control (T0, 90% water), drought stress alone (T1, 30% water), 35 mL PGPR/kg soil (T2, 30% water), 2.5%/kg soil BC (T3, 30% water), and a combination of BC and PGPR (T4, 30% water). Combined PGPR and BC strongly mitigated the negative effects of drought by improving the shoot length (37.03%), fresh biomass (52%), dry biomass (62.5%), and seed germination (40%) compared to the control. The PGPR + BC amendment treatment enhanced physiological traits, such as chlorophyll a (27.9%), chlorophyll b (35.3%), and total chlorophyll (31.1%), compared to the control. Similarly, the synergistic role of PGPR and BC significantly (*p< 0.05*) enhanced the antioxidant enzyme activity including peroxidase (POD), catalase (CAT), and superoxide dismutase (SOD) to alleviate the toxicity of ROS. The physicochemical properties (N, K, P, and EL) of the soils were also enhanced by (85%, 33%, 52%, and 58%) respectively, under the BC + PGPR treatment compared to the control and drought stress alone. The findings of this study have suggested that the addition of BC, PGPR, and a combination of both will improve the soil fertility, productivity, and antioxidant defense systems of barley under drought stress. Therefore, BC from the invasive plant *P. hysterophorus* and PGPR can be applied to water-deficient areas to improve barley crop production.

## 1 Introduction

Barley *(Hordeum vulgare* L.) is an important winter cereal crop of the Poaceae family, grown in the arid and semi-arid regions of West Asia and North Africa (Hafez et al., 2020), and is a well-known quality food crop worldwide (Hafez et al., 2016). Approximately 25% of the world’s barley crop is malted or used as human food and 75% is used as animal feed (El-Hashash and El-Absy, 2019: Newton et al., 2011). Barley crops cover an area of 0.047 billion ha worldwide, and the cultivation of this plant produces approximately 0.147 billion tons (Scheitrum et al., 2020). It has a short life cycle, is resistant to saline and drought stress, and is ranked as the 4^th^ most widely grown cereal crop after wheat, rice, and maize (Akhtar et al., 2021). Barley is a suitable crop for areas where irrigation is limited and is highly tolerant to many other biotic and abiotic stresses (Wiegmann et al., 2019), making it suitable for cultivation in challenging environments (Feiziasl, 2022).

One of the life threating and environmental stresses that effects plant growth, soil quality, and water availability is known as drought stress (Kilic, 2014). Drought presents a severe threat to humans and agriculture, with approximately 53 million people worldwide being affected by limited water availability (Elakhdar et al., 2022). Water scarcity and high temperatures are worldwide issues affecting the survival of agricultural crops and sustainable food production (Mertz-Henning et al., 2018). Severe drought stress results in inhibition of photosynthesis, metabolic disturbance, chlorosis, necrosis, and tissue damage (Jaleel et al., 2009). Drought stress reduces transpiration in plants, which is required for nutrient uptake from the soil and results in growth retardation and development (Çakmakçi et al., 2007). Plant turgidity, metabolism, and osmoregulation are inhibited in response to water-deficit conditions, leading to decreased crop production and development (Gordon et al., 2019). Crop response to drought stress is a critical issue. Therefore, there is a strong need to develop new methods to increase tolerance mechanism in plants (Ashoub et al., 2015). Two approaches are currently used to reduce the negative effects of drought stress on plant growth, that is, the application of plant growth-promoting rhizobacteria (PGPR) and biochar (BC) to soils. Both approaches are fast-growing, eco-friendly, and inexpensive in enhancing plant productivity under abiotic stress (Ullah et al., 2020).

Plant growth-promoting rhizobacteria are endophytic bacteria present in plant roots that promote plant growth and interact positively with plant and soil microorganisms (Forni et al., 2017). PGPR colonize the rhizosphere of plants to increase plant growth *via* direct or indirect mechanisms (Santoyo et al., 2021). Direct mechanisms include nutrient acquisition such as nitrogen fixation, P solubilization, and production of phytohormone. Meanwhile, indirect mechanisms inhibit the function of other pathogenic organism through biocontrol agents (Frampton et al., 2012) PGPR acts as a fertilizer for crop protection, soils structure, decomposition of organic matter and recycling of essential nutrients under abiotic stress, that is, drought stress (Kasim et al., 2016; Gouda et al., 2018). Under water deficit condition, PGPR promote root development in the soils (Kasim et al., 2016). In maize, it has been found that the application of PGPR is effective for plant growth and protection under drought stress (Khan et al., 2021), with positive roles in increasing crop production and yield (Akhtar et al., 2021). Akhtar et al. (2015) reported that PGP and BC enhanced physiological and reproductive responses in maize under saline conditions.

In agriculture, biochar (BC) is also known as “black gold” because it is produced from fossils and dead plant tissues. It increases essential minerals in the soil, including nitrogen (N), sulfur (S), phosphorous (P), carbon (C), and potassium (K) (Sánchez-Reinoso et al., 2020; Ullah et al., 2020). BC is used on farmlands to boost carbon and nitrogen levels (Pérez-Cabrera et al., 2022) and may sequester atmospheric carbon dioxide (CO_2_) (Feng et al., 2021). According to the EPA (2010), approximately 40% of greenhouse gases generated by of the addition of chemical fertilizers to soils can be reduced by adding BC (Schmidt et al., 2014). The highly porous structure and surface area of BC can alleviate the effects of drought because of its water retention capacity owing to adhesion and cohesion forces (Dempster et al., 2012). BC promotes water use efficiency because its structure consists of an oxygen functional group that stores more moisture in the soil (Suliman et al., 2017) Recently, it has been used to increase cowpea plant productivity, nutrient uptake, and antioxidant activity under drought stress (Farooq et al., 2021). Some reports have suggested that BC from *Parthenium hysterophorus* can be used as green muck, compost, and in soil bioremediation to enhance the physiological and biochemical characteristics of plants (El-Naggar et al., 2015; Tefera et al., 2022).

*Parthenium hysterophorus* is native to America, but has become invasive in Asia, Australia, and Africa (Javaid, 2010). *P. hysterophorus* was first introduced to India in 1955 and was later transported to Pakistan (Khan et al., 2012). This weed is among the top 10 plants that are harmful to agricultural systems (Rout and Callaway, 2012), and toxic to humans (Khan et al., 2012). Therefore, the positive management of this weed and biochar production are alternative ways to use it in the environment. However, BC production from this weed could be a useful strategy for controlling environmental stressors in agroecosystems (Masulili et al., 2010; Kumar et al., 2013). Biochar from *P. hysterophorus* can significantly enhance crop productivity and increase soil nutrients (Ahmad et al., 2021). Barley plants were found to be tolerant to salt stress following the application of BC and PGPR (Cardinale et al., 2015). The combined effect of PGPR and BC holds potential for *Brassica napus* in alleviating the toxic effects of drought, directly or indirectly, to promote physiological and biochemical responses (Lalay et al., 2022). Ain et al. (2023) observed a positive effect from *P. hysterophorus* biochar on the growth traits, physicochemical properties, and antioxidant activities of rice and wheat. Therefore, PGPR and BC may interact to enhance plant productivity and tolerance to various stressors. To date, no studies have yet reported the effects of the interactions between PGPR and BC on soil fertility or barley growth productivity. Therefore, the present study was conducted to investigate the effects of BC from *P. hysterophorus* and PGRP on the growth, physiology, and reproductive output of barley under drought stress conditions. We also evaluated the positive effects of PGPR and BC on soil fertility and nutrient uptake by barley under drought stress.

## 2 Materials and methods

### 2.1 Plant materials, soils analysis and treatment

This experiment was conducted at the PMAS Arid Agriculture University, Rawalpindi, Pakistan. Barley seeds were obtained from the Crop Science Institute, National Agricultural Center (NARC), Islamabad, Pakistan. The experimental design was completely randomized, with three replicates. Sodium hypochlorite (5% solution) was used for seed surface sterilization, and then the seeds were washed thoroughly with distilled water two to three times. The sterilized seeds were planted in pots containing soil collected from the botanical garden of the university. The soils were analyzed before and after the experiment using a protocol following the work of Akhtar et al. (2021). This included a suspension of water and soil at a 1:1 ratio, which was stirred and retained for 28 min. One hour later, the pH of the solution was measured using a pH meter.

To determine the total soil organic matter (SOM), 1.2 g of soil was mixed with K_2_Cr_2_O_7_ (10 mL) and 15 mL H_2_SO_4_ (concentrated). After 40 min, 250 mL of deionized water was added along with 8 mL of H_3_PO_3_ and 14 drops of phenolphthalein indicator for color (Ullah et al., 2020). Total nitrogen (N) was determined using the salicylic acid method. Total potassium (K) was determined by mixing 3 g of soil with 30 mL of ammonium acetate and shaking the mixture shaken for 5–8 min. The mixture was then centrifuged at 12000 rpm for 5 min. The supernatant was diluted with ammonium acetate, and readings were recorded on a flame photometer. The Olsen P method was used to determine the (P) (Ullah et al., 2020; Akhtar et al., 2021). The average soil pH ranged from 7 to 7.6, electrical conductivity (EC) 45 dS·m^−1^, total nitrogen in the form of nitrate, and organic content 0.55 mg·kg^−1^, available K 45 mg·kg^−1^, and available P 32 mg·kg^−1^ (**Table 1**).

**Table 1:** Physicochemical characteristics of soils and biochar.

Characteristics of soils	Value	Characteristics of biochar	Value
Texture	Loamy	Ash content (%)	18.5
PH	7.5	PH	9.3
EC (dS^-1^)	45	EC (dS^-1^)	6.45
K(mg/kg^-1^)	45.4	Total N (%)	1.9
N (mg/kg^-1^)	0.55	Total P (g/kg^-1^)	2
P (mg/kg^-1^)	32	Total K (g/kg^-1^)	10
TOM (%)	0.74		

**Figure 1:**
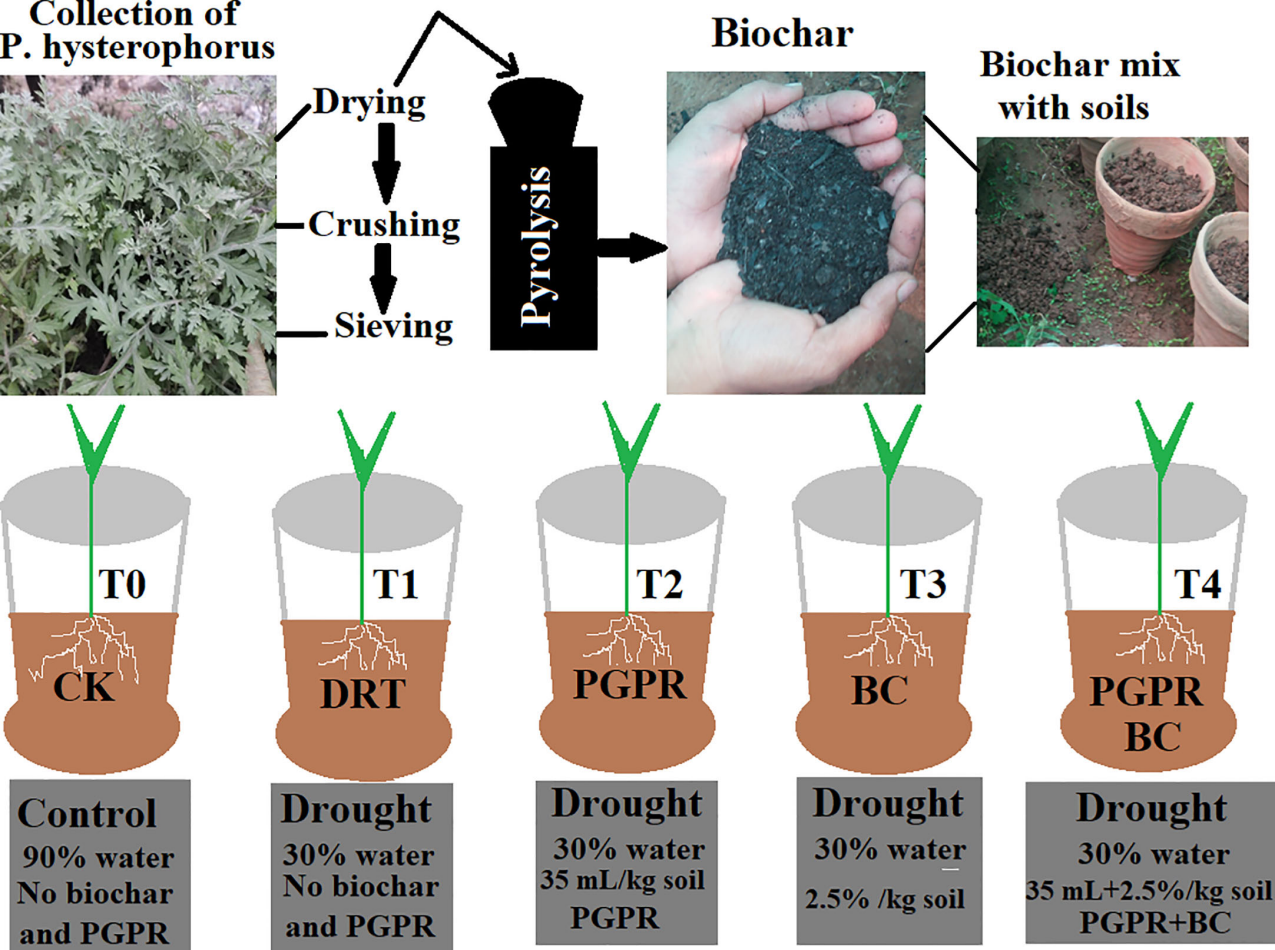
Schematic diagram for preparation of biochar from *P. hysterophorus* by pyrolysis method and detailed experimental design with treatments and control. T0 indicate control, T1 (only drought), T2 (drought and PGPR), T3 (drought and biochar) and T4 (drought combined with biochar and PGPR).

Biochar was extracted from an invasive plant (*P. hysterophorus*) collected from the Bannu district, Slaima Sikander Khail Issaki, Bannu, KPK, Pakistan. Fresh biomass (leaves and stems) of *P. hysterophorus* was cut into small pieces, washed two to three times with distilled water and placed in a dryer for 3–5 d. The dry biomass were placed inside a furnace (350–450 °C) for 60 min and biochar obtained using the pyrolysis method (Mondal et al., 2016; Ain et al., 2023) (**Figure 1**). The physicochemical properties of the prepared biochar are listed in **Table 1**. We used a known drought-tolerant PGPR strain (accession number KC425221, *Serratia odorifera*) that had already been screened in the study by Bangash et al. (2013). The bacterial strain was grown in LB media (Luria–Bertani) and incubated for 3 d at 30 °C. The optical density was measured using a spectrophotometer at 530 nm (BMS VIS), and uniform colony forming units (10^8^ CFU/mL) were obtained for seed inoculation (Ullah et al., 2020).

### 2.2 Study design

Five treatments were used to test the effects of PGPR and BC application on the barley seedlings. A total of 15 pots were used in this study. Three pots were used for each treatment and every pot was filled with 4 Kg soil. 4 kg soil mixed with 2.5% (100 g) ground biochar, and the PGPR strain was added during the inoculum method 35 mL/kg soil (10^8^ CFU/mL), excluding the control and drought stress treatments. Barley seeds (15–20) were sown in each pot, with three replicates per treatment. Plants were watered with tap water and a standard NPK solution was applied as a fertilizer (Hafez et al., 2020). One month later, barley plants were subjected to drought conditions (30% water) for two weeks, except the three control pots (90% water) (Della Torre et al., 2021; Khan et al., 2021). The growth condition of the greenhouse were maintained at 20–25 °C, relative humidity 45–50%, with a controlled light environment at 14/10 h (Akhtar et al., 2021).

The experimental design consisted of 5 treatments × 3 replicates = 15 pots. The different treatments were organized as follows (**Figure 1**) (Tanveer et al., 2023). T0, control (no PGPR or BC); T1 = Drought (no PGPR or BC); T2, 35 mL PGPR/kg soil only; T3, 2.5% BC only; T4, 2.5% BC + 35 mL PGPR/kg soil. After treatment, all the plants were photographed, harvested, and stored at −80 °C for further analysis.

### 2.3 Growth and reproductive traits

For the seed germination assay, the germination percentage of barley plants was calculated using the following equation:


Germination (%)=[total seeds germinated/total number of seeds planted]×100.

Different morphological parameters were measured for each plant, including the root length, shoot length, whole-plant fresh biomass, and whole-plant dry biomass (Rono et al., 2021).

### 2.4 Measurement of electrical conductivity

To measure the membrane stability index (MSI), leaf discs of each plant were placed in 10 mL of distilled water and heated in a water bath at 40 °C for 30 min. The electrical conductivity (EC) was measured for the same sample after being heated to 100 °C for 10 min (C1). The EC was then measured after the sample had been heated (C2). The MSI of each plant was calculated using the following formula (Khan et al., 2021):


M.S.I=[1−(C1/C2)]×100

### 2.5 Assessing the total chlorophyll content

Chlorophyll a, chlorophyll b, and total chlorophyll were assessed for each plant using the method described by Khan et al. (2020) and optimized by Arnon et al. (1949), with slight modifications. Fresh leaves were ground in 10 mL of 100% acetone and were then filtered using Whatman paper to collect the filter extract in a separate test tube. The filtrates were retained at 28 °C for 2–3 d in the dark and the absorbance were checked at 645 nm and 663 nm wavelengths respectively in the spectrophotometer (Thermo Scientific, EVO 60, Germany).

Chlorophyll a, chlorophyll b, and total chlorophyll were determined using the following formula (Khan et al., 2020):


Chlorophyll a=12.7(A663)−2.7(A645), Chlorophyll b=22.9(A645)–4.7(A663)

Total chlorophyll=Chlorophyll a+Chlorophyll b

### 2.6 Antioxidant enzyme activity

To examine the antioxidant enzyme activity for each plant treatment, we followed the protocol of Khattak et al. (2022) with slight modifications. Fresh barley leaves (0.4 g) from each treatment were ground with Tris buffer (NaH_2_PO_4_) and Na_2_HPO_4_ (pH 7) and mixed well before being placed in the centrifuge at 13000 rpm for 17 min at 4 °C. The pellets were removed and the supernatant was then used for enzyme extraction for various assays (POD, CAT, and SOD). The peroxidase (POD) activity was determined spectrophotometrically (Thermo Scientific, EVO 60) at 470 nm, with reaction mixture (1 mL 0.3% H_2_O_2_, 0.95 mL 0.2% guaiacol, 1 mL 50 mM PBS and 100100 µL). The enzyme activity was recorded every 30 s and at least six readings were obtained. The catalase (CAT) activity was assessed as described by Akhtar et al. (2021) with slight modifications. The reaction mixture contained 0.3% H_2_0_2_, 1.9 mL water, and 100 µL enzyme extract mixed together and recorded at 240 nm. For superoxide dismutase (SOD) activity, the protocol of Zulfiqar et al. (2022) was used. The reaction mixture contained 3 mL including PBS buffer (50 mM), nito-blue tetra azolium (70 µM), and methionine (13 mM) was mixed with 0.1 µM (EDTA) + 0.1 mL enzyme extract to form a solution. The absorption was checked at 560 nm using a spectrophotometer (Thermo Scientific, EVO 60).

### 2.7 Statistical analysis

Statistical analysis of the soil physicochemicals were analyzed using one-way ANOVA with Tukey’s test (p< 0.05) (IBMS, Amos, 21). The student’s t-test (p< 0.05) was used for pairwise comparisons among treatments with the control. All experimental data were obtained in triplicates. Significant differences at different p values (p< 0.05, one asterisk; p< 0.01 indicate double asterisk), standard deviations, and mean separations were conducted. Origin Pro 8 was used to design all the figures in this study.

## 3 Results

### 3.1 Physical and chemical properties of post harvested soils

The physical and chemical properties of the soils changed in response to the addition of BC, PGPR, or their combination. The pH and electrical conductivity (EC) of the soils increased with the addition of BC and PGPR (**Table 2**). All the soil nutrients, that is, total N, total P, and total K, significantly increased in the soils treated with BC alone, PGPR alone, and the combination of PGPR and BC. However, these essential nutrients decreased under drought conditions compared with the control (**Table 2**). The total organic matter (OM) of the post-harvested soils also showed a similar trend, because it significantly increased with the addition of biochar and PGPR but decreased under drought stress alone.

**Table 2:** Physicochemical properties of the post harvested soils.

Treatments	pH	EC (dsm^-1^)	NO_3_-N (mg/kg^-1^)	P (mg/kg^-1^)	K (mg/kg^-1^)	OM (mg/kg^-1^)
CK	7.25 b	0.17 b	2.75 c	34 c	45 c	0.48 c
DR	6.55 c	0.12 c	1.80 d	25 d	30 d	0.30 d
BC	7.40 ab	0.22 a	4.99 ab	50 a	50 b	1.9 ab
PGPR	7.20 b	0.2 ab	3.65 b	43 ab	52 ab	1.45 b
BC+PGPR	7.58 a	0.27 a	5.1 a	52 a	60 a	2.3 a

Different letters indicate significantly differences among treatments at P< 0.05 (Tukey’s test).

### 3.2 Effects of PGPR, biochar, and drought on growth and reproductive traits in barley

**Figure 2:**
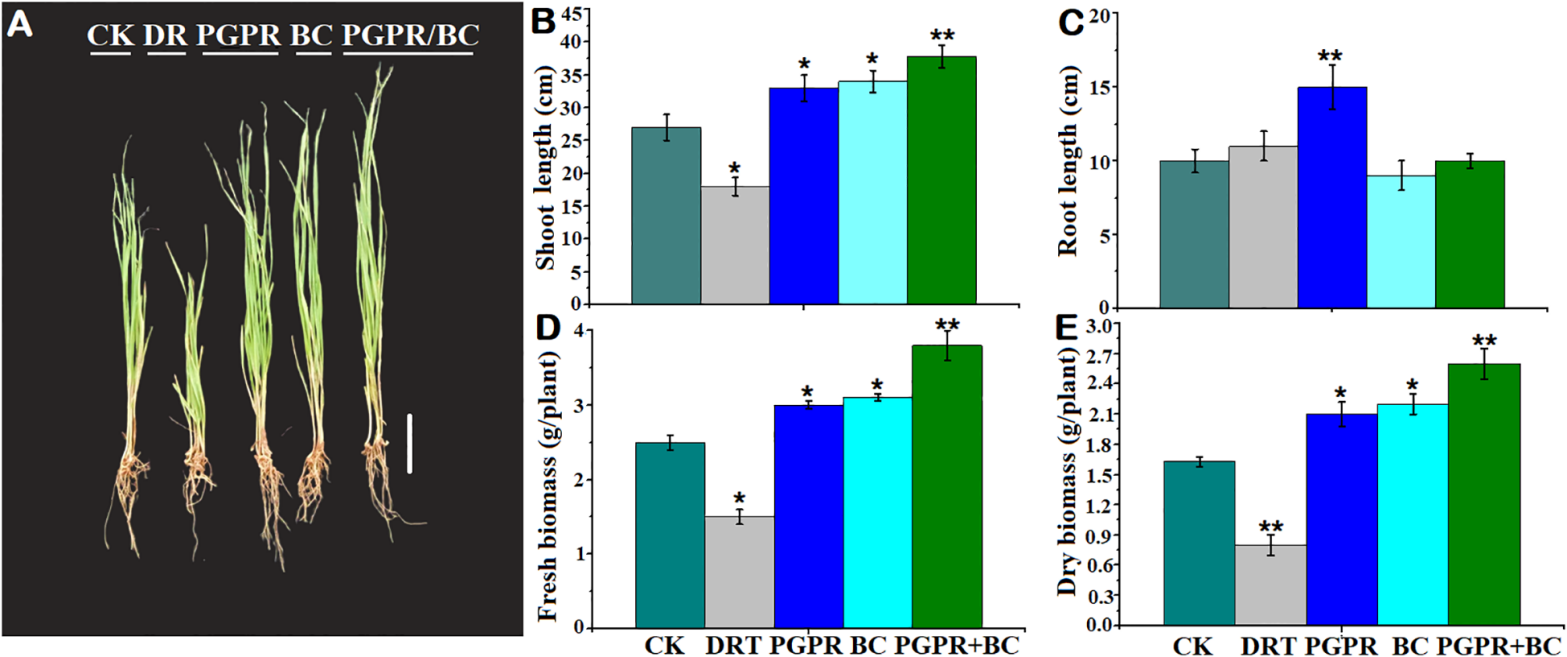
Growth response of barley with or without plant growth promoting rhizobacteria (PGPR), biochar (BC) and combined PGPR and BC (PGPR+BC) under drought (DRT) stress. **(A)** Plant phenotype, **(B)** Shoot length, **(C)** Root length, **(D)** Fresh biomass, **(E)** Dry biomass. The bars represent the standard deviation with 3 replicates. A single asterisk represents significant differences at *p*< 0.05, while two asterisks represent significant differences at *p*< 0.01 compared to the control (student t test).

The growth of barley significantly increased with the amendment of PGPR, BC, and combined BC + PGPR compared to the control. However, it significantly decreased under drought alone (**Figure 2A**). Shoot length in barley was significantly elongated under the PGPR, BC, and PGPR + BC treatments, which was 37.03% higher in the PGPR + BC treatment than the control (**Figure 2B**). The shoot length of barley was significantly inhibited by drought treatment in the absence of PGPR and BC. The root length of the barley plants was substantially higher (35%) under the treatment with PGPR alone treatment compared to the control. However, there was no significant difference in the other treatments (**Figure 2C**).

Fresh biomass of the barley plants was significantly (*p< 0.05*) higher than the control by 20%, 24%, and 52% in the PGPR, BC, and PGPR + BC treatments, respectively. However, it decreased by 20% under drought stress alone (**Figure 2D**). A similar trend was observed for plant dry biomass, which was significantly higher under PGPR + BC treatment by 62.5% compared to the control. However, it decreased under the treatment with drought stress alone (**Figure 2E**).

**Figure 3:**
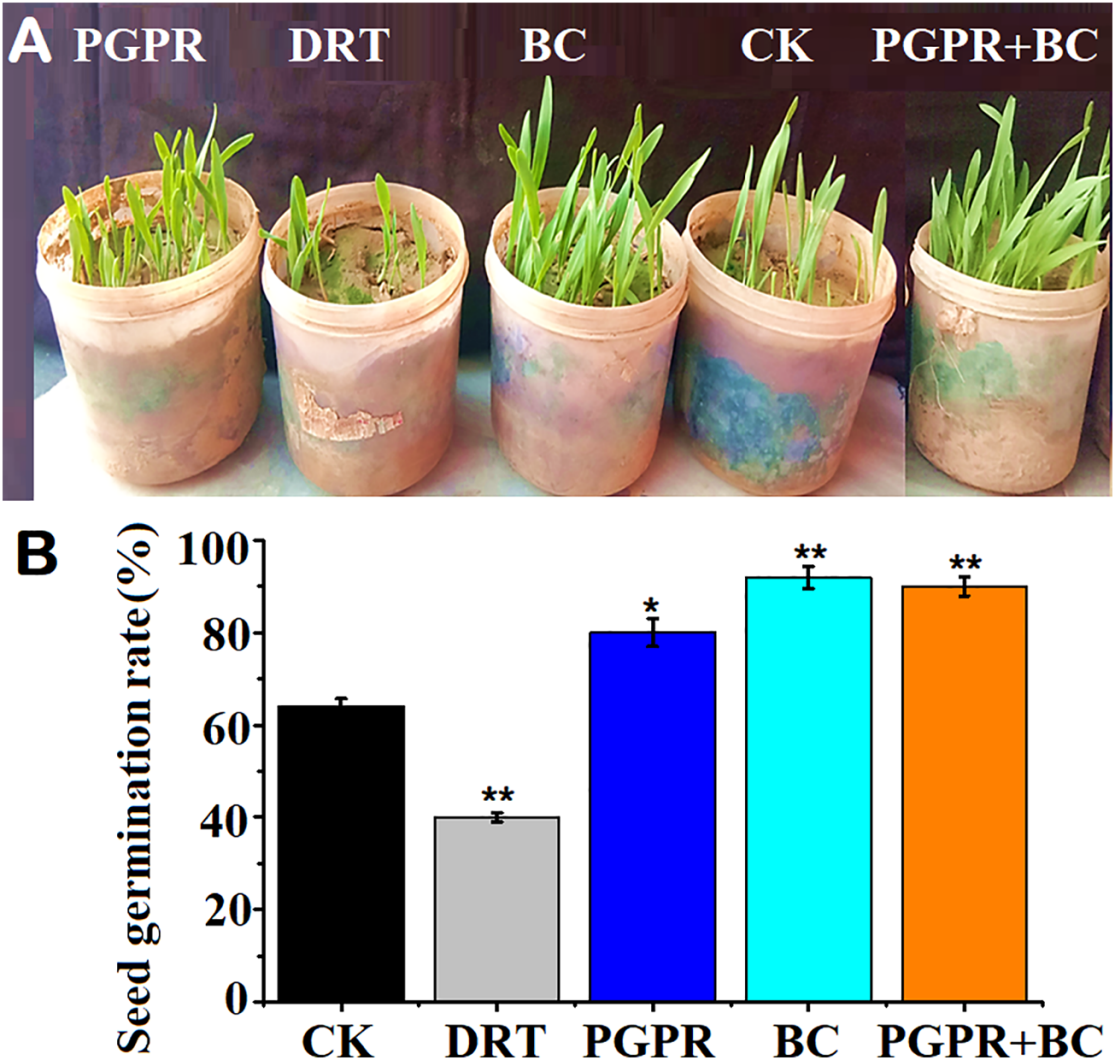
Seed germination assay of barley with or without plant growth promoting rhizobacteria (PGPR), biochar (BC) and PGPR+BC under drought stress. **(A)** Germinated seeds, **(B)** seed germination rates. The bars represent the standard deviation with 3 replicates. A single asterisk represents significant differences at p < 0.05, while two asterisks represent significant differences at p < 0.01 compared to the control (using student t test).

The seed germination rate in the soils treated with BC was significantly higher by 42.4% (*p< 0.05*) that of the control (**Figure 3**). The second-highest seed germination rate was recorded in the BC combined with PGPR treatment (**Figure 3**). The seed germination rate in the PGPR alone treatment was slightly higher than that of the control (**Figure 3**). Under drought conditions, the seed germination rate was significantly lower than that of the control (*p< 0.05*, **Figures 3A, B**: Supplementary Data).

### 3.3 Effects of PGPR, biochar, and drought on physiological traits in barley

**Figure 4:**
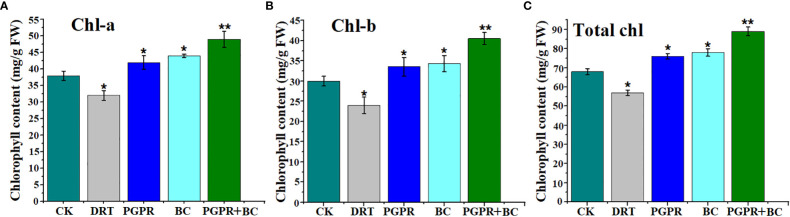
Chlorophyll content of barley plants with or without plant growth promoting rhizobacteria (PGPR), biochar (BC) and PGPR together with BC under drought stress. **(A)** Chlorophyll-a, **(B)** Chlorophyll-b, **(C)** total chlorophyll. The bars represent the standard deviation with 3 replicates. A single asterisk represents significant differences at *p*< 0.05, while two asterisks represent significant differences at *p*< 0.01 compared to the control (using student t test).

The chlorophyll-a (chl-a) content of the plants was significantly lower in the drought treatment than in the control. Meanwhile, the amendment of biochar and PGPR significantly enhanced chlorophyll-a in barley compared with the control (**Figure 4A**). A similar trend in chlorophyll b (chl-b) content was observed under the BC, PGPR, and combined BC + PGPR treatments compared to the control. However, it significantly decreased under drought stress alone (**Figure 4B**). The total chlorophyll content of the plants was significantly higher under the PGPR, BC, and PGPR + BC treatments than that of the control by 10.6%, 15.6%, and 31.1%, respectively. However, it decreased by 16.5% under the treatment with drought stress alone (**Figure 4C**).

### 3.4 Total antioxidant activity in barley

**Figure 5:**
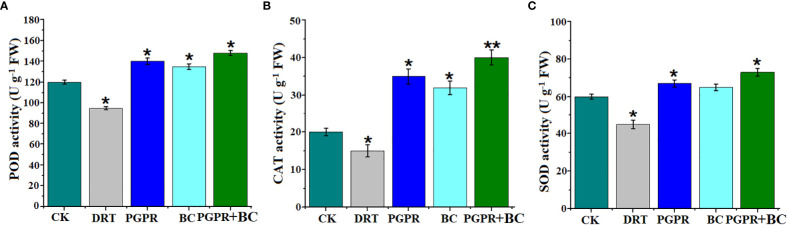
Antioxidant enzyme activity of barley plants in the control (well-watered), drought (alone), plant growth promoting rhizobacteria (PGPR with drought), biochar (BC with drought) and with PGPR and BC combined (with drought). **(A)** Peroxidase activity, **(B)** catalase activity, **(C)** superoxide dismutase activity. The bars represent the standard deviation with 3 replicates. A single asterisk represents significant differences at *p*< 0.05, while two asterisks represent significant differences at *p<* 0.01 compared to the control (using student t test).

The antioxidant enzymatic activities (POD, CAT, and SOD) of barley significantly increased in response to most treatments. However, they decreased significantly by 20%, 25%, and 25% respectively under conditions of drought stress alone compared to the control (**Figures 5A–C**). The highest POD, CAT, and SOD values were recorded (23.3, 100%, and 21%) in the combined BC + PGPR drought stress treatment (**Figure 5**). This suggests that BC and PGPR promote antioxidant enzyme activity to alleviate ROS toxicity.

### 3.5 Plant uptake activity

**Figure 6:**
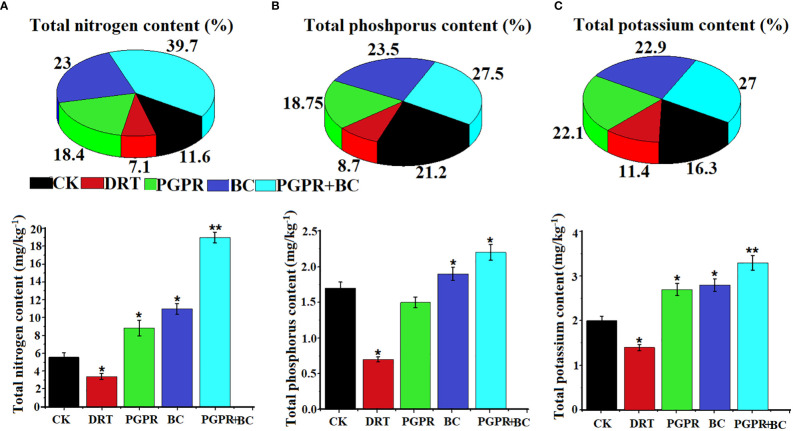
Nutrient uptake activity of barley plants in the control (well-watered no PGPR and BC), drought (alone), plant growth promoting rhizobacteria (PGPR with drought), biochar (BC with drought) and PGPR with BC (with drought). **(A)** Total nitrogen content, **(B)** total phosphorus content, and **(C)** Total potassium content. The bars represent the standard deviation with 3 replicates. A single asterisk represents significant differences among treatments at *p*< 0.05, while two asterisks represent significant differences at *p<* 0.01 compared to the control (using student t tests).

The uptake of nitrogen (N), potassium (K), and phosphorus (P) by barley under drought stress was examined. Soil nutrient uptake activities of barley plants were significantly enhanced under BC, PGR, and the combination of BC + PGPR (**Figure 6**). The highest (39.7%) total N uptake was recorded in the PGPR and BC combined treatment, whereas the lowest (7.1%) occurred under the drought stress treatment (**Figure 6A**). The total P content was highest under the PGPR + BC treatment (27.5%), and the second highest was observed under the BC alone treatment (23.5%). These were the only treatments higher than the control (**Figure 6B**). The total K uptake was highest (27%) under the BC + PGPR treatment and lowest (11.4%) under the treatment with drought stress (**Figure 6C**).

## 4 Discussion

The phenotypic, biological, agronomic, and physiological properties of plants are strongly affected by drought stress (Mertz-Henning et al., 2018). Water deficit is one of the global agricultural issue to restricts crop productivity and development, including plant growth, nutrient uptake, and antioxidant activity (Laird et al., 2010). Therefore, it is necessary to develop effective approaches for tolerance and resistance to drought stress. PGPR and BC activities can generally improve plant tolerance and productivity (Akhtar et al., 2021). PGPR can promote plant growth either directly or indirectly, that is, the direct mechanism of PGPR uses resource acquisition including essential minerals, ACC deaminase, auxin, nitrogen fixation, and phosphorus solubilization. Meanwhile, the indirect mechanism inhibits the function of other pathogenic organisms through biocontrol agents and cell wall degradation by enzyme production (Frampton et al., 2012). Different strains of PGPR have been proven to enhance plant growth, for example *Azosprilillum*, *Bacillus*, and *Serratia* (Akhtar et al., 2021: Bangash et al., 2013). BC is another strategy used for enhancing plant growth and development because it stores more water and increases nutrient uptake, which promotes the growth of the rhizosphere (Khan et al., 2021).

In the present study, we found that the combined treatment with PGPR (*Serratia odorifera*) and biochar (*P. hysterophorus)* enhanced soil organic content, total nitrogen, total phosphorus, and total electrical conductivity under drought stress. This was because BC has ash content, nutrient content, and increased holding capacity. These results have suggested that biochar and PGPR have a potential positive effect on soil fertility. Algal biochar and PGPR enhanced soil quality and organic matter, as well as total essential minerals. This is because algal BC has organic carbon that may enhance the bacterial population in the rhizosphere, which involves soil nitrification to promote total soil minerals (Ullah et al., 2020). Soil organic matter and essential minerals increase in soils because of the porous structure and ash content of the biochar, which enhanced the absorption ability of different cultivator (Yu et al., 2023). Ain et al. (2023) found that biochar from *P. hysterophorus* enhanced the organic matter and total nitrogen content in soils where rice plants were grown under drought and saline conditions. The potential effects of biochar on soil fertility, organic matter, and important minerals have been widely reported (Tak et al., 2013; Awad et al., 2019; Eissa, 2019).

In this study, we observed a positive correlation between PGPR and BC with increasing barley growth. The barley phenotypes were more vigorous when treated with BC, PGPR, or a combination of both compared to the control. Meanwhile, drought stress alone severely impacted the plants. The shoot length was significantly higher under the PGPR and BC treatments than under the control, which decreased the negative effects of drought stress alone (**Figure 2**). Therefore, it can be concluded that combining PGPR with BC significantly enhances plant growth under drought conditions. This result is in line with the findings of a previous study showing that the phenotypes of rapeseed improved when treated with biochar to promote growth under water-deficit conditions (Khan et al., 2021). Tomato plant phenotypes and shoot and root lengths significantly improved after amendment with biochar and PGPR, which significantly enhanced plant resistance to drought (Wang et al., 2021). Awad et al. (2019) highlighted that barley growth and development were enhanced after amendment with two different concentrations of biochar compared with the control treatment. In the present study, the root length was considerably longer under drought stress and PGPR treatments compared to BC. This suggested that water deficit could induce roots to grow deeper into the soils to enhance water uptake due to inoculation of the PGPR *Serratia odorifera* strain. This has the capability to form indole acetic acid (IAA) which is capable of increasing stimulation of amino cyclopropane carboxylic acid synthase that converts toxic ethylene to amino-cyclo-propane carboxylate (ACC). ACC may help convert the ethylene hormone to α-ketobutyrate and ammonia forms in the rhizosphere. Barley roots can become substantially elongated after decreased stimulation with ethylene (Bangash et al., 2013). BC from P. *hysterophorus* acts as a bodyguard for PGPR to enhance root proliferation. This provides essential nutrients and adjusts the water levels owing to its high surface area, increasing the electrolyte capacity and water-holding capacity (Hussain et al., 2021). This is supported by another study in which maize roots were elongated under drought stress when treated with PGPR and BC compared to the control (Ullah et al., 2020).

The seed germination rate of barley was significantly higher under biochar and the combination of BC and PGPR under drought stress (Saeed et al., 2022). These findings suggest that P. *hysterophorus* biochar would help to improve the barley plants during the early stages of seed germination under water-deficient conditions. This is because it may increase the soil essential nutrients (N, P, and K) owing to the porous and negatively charged structure to decrease soil leaching and enhance soil mineralization by directly aborting plant roots from the soil (Ain et al., 2023). It may also activate the metabolic system in the soils to activate PGPR inside the root zone. In this study, the PGPR strain may have increased the seed germination rate by increasing the production of essential growth hormones, that is, auxin and cytokinin, to enhance the production of enzyme activity. In this context, amylase can help to promote the levels of starch (Akhtar et al., 2021). The fresh and dry biomass of barley was significantly higher when treated with biochar and PGPR. Danish et al. (2020), reported the positive effects of ACC deaminase PGPR on maize for enhancement the maize development and biological response. Growth rate, chlorophyll content, and enzymatic activity of potato plants significantly increased with the inoculation of PGPR o resist the water scarcity condition (Batool et al., 2020). This is in line with the findings of the present study. Biochar from *P. hysterophorus* has been found to significantly improve seed germination, shoot height, and root length in maize plants (Kumar et al., 2013). This suggesting that there is some beneficial use for some invasive plant species in agriculture.

Plant physiological responses are important during abiotic stress (Khan et al., 2020) and chlorophyll levels can be used as an indicator for various abiotic stress in the plant (Wang et al., 2019). Water deficit condition, negatively affects the plant growth and physiological response (Mertz-Henning et al., 2018). However, it was previously found that the chlorophyll content in barley significantly increased under drought stress after amendment with BC (Tak et al., 2013). This is similar to the observations in the current study (**Figure 4**). Chlorophyll a and b were significantly higher in tomato plants following the application of PGPR (Batool et al., 2020), as well as in barley plants treated with BC (Awad et al., 2019). This suggests that BC and PGPR may benefit plant physiological responses. Rice plants have significantly enhanced chlorophyll content owing to the presence of PGPR, which promotes photosynthetic efficiency (Hafez et al., 2016). Our results have indicated that combining PGPR-tolerant strains with BC from *P. hysterophorus* enhances the ability of barley plants to survive under water-deficit conditions and can maintain photosynthetic pigments. The chlorophyll a, b and total chlorophyll content of the current proposed study significantly higher under BC and PGPR treatments because Chlorophyll is directly linked to the nitrogen content in the leaves. After the amendments of PGPR and BC, the photosynthetic rate in barley plants was enhanced because of increased N uptake activities from the soil *via* the xylem to close the stomata and prevent water loss under drought stress (Lalay et al., 2021). PGPR can prevent net photosynthetic activities by producing cytokinin hormones to close the stomata and enhance leaf cell division in the early growth stage. This then leads to an increase in the number of vascular bundles to promote leaf area (Sivasakthi et al., 2013; Hönig et al., 2018). Application of PGPR and BC may improve root growth, which, in turn, improves nutrient intake and ultimately increases photosynthetic activity.

The crop antioxidant defense system is triggered under water deficit conditions to absorb reactive oxygen radicals, which potentially affect numerous organelles in plants (Zhang et al., 2021). Antioxidant enzymes in barley plants may potentially mitigate the negative effects of drought stress by scavenging reactive oxygen species (ROS) and preventing cell damage (Gupta et al., 2015). The present findings have shown that catalase (CAT), peroxidase (POD), and superoxide dismutase (SOD) were significantly increased under drought stress following the addition of PGPR and BC. This alleviated the negative effects of ROS owing to PGPR-activated self-protective systems and the production of enzymes inside the plant (**Figure 5**). Our results are in line with those of other studies that found increased CAT, POD, and SOD activities under drought stress (Abid et al., 2019). This suggests that plants treated with PGPR and BC may produce ROS that protect the photosynthetic apparatus (Hafez et al., 2020). Barley plants showed decreased toxicity caused by reactive oxygen species after amendment with BC and chitosan, which boosted antioxidant enzyme activity. The findings of the current study are in line with those of Khan et al. (2021), who observed a positive role from biochar in reducing the toxic effects of drought stress supported by SOD, CAT, and POD enzyme activities.

Addition of biochar and PGPR increases the plant uptake of essential nutrients from the soil (Abdipour et al., 2019). Nutrient uptake in barley plants significantly increased after treatment with a range of biochar concentrations under drought stress (Awad et al., 2019). In the present study, we found that the uptake of total nitrogen, total potassium, and total phosphorus was significantly enhanced after amendment with biochar, PGPR, and a combination of both. This is because this PGPR strain has the capability to take up more nutrients from the soil by a direct mechanism to release the organic compound responsible for nutrient availability (Ullah et al., 2020). Therefore, PGPR and BC can enhance the uptake of essential nutrients under drought conditions. Yu et al. (2023) hypothesized that nitrogen uptake activity increases because of plant growth bacteria associated with the nitrogen cycle helping plants resist abiotic stress. In contrast, plants treated with biochar have enhanced uptake of total nitrogen, phosphorus, and potassium because they have negatively charged ions and cohesive forces that boost mineralization in soils (Laird et al., 2010). Mahmood (2022) also observed that more phosphorus was absorbed by plants treated with PGPR under high stress conditions.

## 5 Conclusion

The results of this study have shown that the positive role of the PGPR drought strain (*Serratia odorifera*) and BC from *P. hysterophorus* mitigated the negative effects of drought stress by improving plant growth, plant biomass, and seed germination. The combination of PGPR and BC significantly enhanced chlorophyll content and activated the antioxidant defense system in barley to resist drought stress. Furthermore, they improved the uptake of essential nutrients, that is, N, P, and K, as well as soil organic matter. Therefore, biochar and PGPR could play a key role in alleviating the negative effects of abiotic stress on agricultural crops. From this perspective, the application of BC and PGPR to other plants under different stress conditions should also be explored.

## Data availability statement

The original contributions presented in the study are included in the article/**Supplementary Material**. Further inquiries can be directed to the corresponding authors.

## Author contributions

Conceptualization: IK, FG, GL and D-LD. Funding acquisition: GL and D-LD. Data curation, investigation, and writing—original draft: FG, IK and SR. Methodology: IK. Writing —review and editing: SR and Z-CD. All authors contributed to the article and approved the submitted version.
